# Analytical Method Development and Chemometric Approach for Evidencing Presence of Plasticizer Residues in Nectar Honey Samples

**DOI:** 10.3390/ijerph17051692

**Published:** 2020-03-05

**Authors:** Ivan Notardonato, Sergio Passarella, Giuseppe Ianiri, Cristina Di Fiore, Mario Vincenzo Russo, Pasquale Avino

**Affiliations:** Department of Agriculture, Environmental and Food Sciences, University of Molise, via De Sanctis, I-86100 Campobasso, Italy; ivan.notardonato@unimol.it (I.N.); sergio.passarella@studenti.unimol.it (S.P.); g.ianiri@studenti.unimol.it (G.I.); c.difiore@studenti.unimol.it (C.D.F.); mvrusso@unimol.it (M.V.R.)

**Keywords:** honey, plastics, phthalates, bisphenol A, UVA-DLLME, GC-IT/MS, specific migration limit, chemometrics, principal component analysis

## Abstract

Over the years, anthropogenic sources have increasingly affected food quality. One of the most sensitive and nutritional matrices affected by chemical contamination is honey, due to the use of acaricides. Recently, the attention has moved to the presence of phthalates (PAEs) and bisphenol A (BP-A), molecules present in plastic materials used both in the production phase and in the conservation of honey. In this study, an analytical method for the simultaneous determination of PAEs (dimethyl phthalate DMP, diethyl phthalate DEP, diisobutyl phthalate DiBP, dibutyl phthalate DBP, bis(2-ethylhexyl) phthalate DEHP, and di-n-octyl-phthalate DnOP) and BP-A was developed. The extraction technique is the ultrasound-vortex-assisted dispersive liquid–liquid microextraction (UVA-DLLME), using 150 µL of toluene as an extraction solvent, followed by the gas chromatography coupled with ion trap mass spectrometry analysis (GC–IT/MS). The developed method is sensitive, reliable, and reproducible: it shows high correlation coefficients (R > 0.999); limits of detection (LODs) less than 11 ng·g^−1^; limits of quantification (LOQs) less than 16 ng·g^−1^; repeatability below 3.6%, except BP-A (11.6%); and accuracy below 4.8%, except BP-A (17.6%). The method was applied to 47 nectar honey samples for evidencing similarities among them. The chemometric approach based on Hierarchical Cluster Analysis and Principal Component Analysis evidenced some similitudes about sample origin as well as marked differences between PAE and BP-A sources.

## 1. Introduction

Honey, always considered “The Food of the Gods”, is a natural sweet substance that bees (*Apis mellifera*) produce from the nectar of plants which they forge, transform, combine with their own specific substances, deposit, dehydrate, store, and let mature in the honeycombs of the hive [1]. Honey has multiple properties, the first of which is certainly nutritional. It is very important in children’s and athletes’ diets due to the absence of potentially harmful artificial sugars. There are also beneficial properties, especially for the respiratory tract (decongestant and calming of coughs) and gastroenteric apparatus [2] as well as antioxidant properties due to the presence of enzymatic antioxidants [3].

In the beekeeping sector, we have witnessed in recent years the invasion of the market with non-EU produced honey not suitable for consumption in the EU, because they come from hives treated with pesticides that have been prohibited in the EU for years now. In this regard, European legislation allows the placing on the market of honey intended for human consumption which “as far as possible must be free of organic and inorganic substances extraneous to its composition” [1]. In fact, the honey produced by *Apis mellifera* is subject to possible contaminated residues used for the fight against parasites such as *Varroa distructor* [4,5,6,7]. Both through the pollen and the nectar foraged from flowers, as well as the water used, bees collect daily the residues of contaminants present in the environment and themselves carry out a first concentration step. Often, precisely for these reasons, the bee products and, even if to a lesser extent, the honey, are considered indicators of environmental contamination [8,9,10,11]. Among the most important food contaminants, phthalates (PAEs) and bisphenol A (BP-A) play a really important role (Table 1).

Phthalates, ubiquitous substances, are esters of phthalic acid, essentially used in the production of plastics to which they give flexibility and resilience [12,13]. The presence of these molecules in plastic polymers (e.g., polyvinyl chloride, PVC) facilitates the flow of monomers, making the material soft and moldable even at room temperature. The possible alarm is because PAEs are not chemically linked to the plastic polymer and, due to thermal and mechanical stress, can also be released in polar matrices such as water [13]. PAEs can come into contact with the human body (by ingestion, inhalation, or contact), generating a health risk. Foods can be contaminated during growth, production, processing, or packaging; fat matrix foods are most at risk. The effects of these molecules on human health have been controversial for years. In particular, low acute toxicity but a worrying chronic toxicity on rats has been highlighted which has led to the development of liver cancer and teratogenicity. However, this symptomatology was not subsequently found in the experimentation carried out on non-human primates [14]. Alterations of the hormonal system with contrasting effects (endocrine disruptors) have been highlighted both on rats and on primates [15,16,17,18]. Finally, it should be considered that plasticizers such as phthalates are largely involved in packaging process [19,20] and have been found in different food matrices [21,22,23,24]. The other very interesting compound is BP-A, an organic synthetic compound, poorly soluble in water (0.344% at 83 °C), used for the production of epoxy resins and polycarbonates for food packaging; it can also be present in water bottles [25,26,27,28,29] as well as in the internal linings of cans. Based on scientific studies, it can be said that BP-A can pose a risk to the health of the consumer, above all due to its cytotoxicity [30,31]. Containers unfortunately, the knowledge of the toxicokinetics is still incomplete and interventions are being considered to eliminate this compound from the human body [32]. A recent study verified the relative concentration of BP-A in three body fluids: blood, urine, and sweat. The BP-A concentration was higher in sweat than in urine and blood; BP-A was found in sweat even when it was not present in the other two body fluids. This implies that induced sweating can be an intervention to facilitate the elimination of this compound and also that the analysis of sweat can be placed as an indicator of contamination [33]. The consumption of canned food by consumers found a 1600% increase in the concentration of BP-A in the urine compared to glass containers. After taking these drinks, blood pressure was recorded again, which recorded a significant and systematic increase in values; the contamination of the human body by BP-A causes an increase in blood pressure [34]. A study, through cross-analysis between the presence of BP-A in the urine and the percentage of fat mass, found that contamination with BP-A is responsible for a higher incidence of obesity and an abnormal girth-height ratio in children [35].

In the absence of legal limits, specific migration limits (SMLs) are applicable to food contact materials containing food additives or polymer production aids that have the potential of leaching into food. It is the maximum amount of substance permissible per kg of food. SMLs have been set up by the Commission Regulation (EU) in regulations No 10/2011 for some PAEs [36] and No 213/2918 for BP-A [37] (Table 1).

The possible presence of PAEs and BP-A in this matrix is not only related to (raw) honey, but also to the remaining production processes which may involve direct contact with unsuitable plastic. In addition, in recent years, the use of plastic honeycombs has spread to reduce the risk of melting the wax itself, with consequent loss of the crop, during the hottest summer seasons. Furthermore, even if marginal, single-dose, it should be considered that plastic packages may be left under the sun for long periods. Ultimately, the critical point is certainly the type and quality of plastic used.

From an analytical point of view, the determination of PAEs and BP-A content is not trivial: the papers regarding their analysis are increasing in these last decade. Specifically, just one paper deals with the determination of PAEs/BP-A in honey samples [22], whereas different papers deal with the analysis of such compounds in human fluids. Recently, three interesting papers were published in this field. Babu-Rajendran et al. [38] investigated the PAE levels in human urine using a GC–MS analysis: limits of quantification (LOQs) range between 0.8-3.9 ng mL^−1^, recoveries between 99% and 104%, and precision below 7.8 (calculated as relative standard deviation, RSD). Pinguet et al. [39] analyzed 22 metabolites of bis(2-ethylhexyl) phthalate (DEHP) in urine by means of turbulent flow online extraction technology coupled with high-performance liquid chromatography–tandem mass spectrometry (HPLC–MS/MS): the analytical validation allowed LOQs to reach 0.01 to 0.1 ng mL^−1^, accuracy between 86% and 117% and interday and intraday precisions < 20%. Finally, Wang et al. studied a multiresidue method for determining 36 endocrine disrupting chemicals, including 8 bisphenols and 14 phthalates, in human serum [40]. They compared Liquid-Liquid Extraction (LLE) and Solid Phase Extraction (SPE) followed by ultraperformance liquid chromatography coupled to tandem mass spectrometry (UPLC–MS/MS): they obtained recoveries ranging between 45.8% and 120% with LOQs between 0.002 and 0.532 ng mL^−1^ and intraday (0.1%–12.7%) and interday (0.2%–13.3%) calculated as RSD.

The aim of this paper is to develop a fast, sensitive, and reproducible method for the analytical determination of phthalates and bisphenol A in honey matrix. The determination of these molecules shows considerable difficulties mainly related to their low concentration, which is difficult to determine even for very sensitive instruments. To overcome this problem, it is necessary to develop analytical methods that provide for extraction and preconcentration steps. In this paper, dispersive liquid–liquid microextraction (DLLME), slightly modified, is used as the extraction technique, followed by gas chromatography combined with ion trap mass spectrometry (GC–IT/MS) as the analytical procedure. The method developed has been validated, all the analytical parameters influencing the extraction efficiency of the analytes are studied and discussed. Forty-seven nectar honey samples, sampled in the Central Italy, are investigated for determining the concentration of plastic residues. A chemometric approach is applied for identifying the clusters and the possible unique source of the considered compounds.

## 2. Materials and Methods 

### 2.1. Chemicals and Reagents

Standards of PAEs investigated in this study, such as dimethyl phthalate (DMP; C_10_H_10_O_4_), diethyl phthalate (DEP; C_12_H_14_O_4_), diisobutyl phthalate (DiBP; C_16_H_22_O_4_), dibutyl phthalate (DBP; C_16_H_22_O_4_), bis(2-ethylhexyl) phthalate (DEHP; C_24_H_38_O_4_), di-n-octyl-phthalate (DnOP); C_24_H_34_O_4_), and bisphenol A (BPA); C_15_H_16_O_2_), were obtained from Sigma-Aldrich (Milan, Italy). In Table 1, analytical data (i.e., Chemical Abstracts Service (CAS) number, molecular weight (MW), selected ion monitoring (SIM), octanol–water partition coefficient (K_ow_)) are reported. *n*-Hexane, *n*-heptane, *iso*-octane, ethyl acetate, and toluene were of pesticide grade (Carlo Erba, Milan, Italy), whereas sodium chloride (Carlo Erba) was of analytical reagent grade. A standard solution of phenanthrene 10 µg mL^−1^ (C_14_H_10_; LabService Analytica, Anzola Emilia, Bologna, Italy) was added as internal standard (I.S.) to each sample before being processed.

Solutions of each phthalate and bisphenol A were prepared at concentrations of 1 mg mL^−1^. Further, PAE and BP-A mix solutions at different concentrations (0.005, 0.01, 0.1, 0.25, 0.5, 1.0, 5.0 µg mL^−1^) were prepared by dilution. The solutions were stored in vials at −20 °C.

For avoiding cross-contamination due to reagents, materials, and laboratory equipment, a severe cleaning procedure was performed: the glassware was soaked and washed in acetone, dried at 140 °C for at least 4 h; NaCl was heated for 4 h at 140 °C and kept in a tightly sealed glass vial. For the PAE standard solutions (0.1 mg mL^−1^ of each PAE), absolute ethanol was used.

### 2.2. Ultrasound-Vortex-Assisted Dispersive Liquid–Liquid Microextraction Procedure

The study of this analytical approach (i.e., the ultrasound-vortex-assisted dispersive liquid–liquid microextraction (UVA-DLLME) procedure) can be divided into several phases. The choice of the best extraction solvent among different solvents, such as *n*-hexane (density 0.66 g cm^−3^), *n*-heptane (0.68 g cm^−3^), *iso*-octane (0.69 g cm^−3^), and toluene (0.867 g cm^−3^), is the first step. All the solvents tested had a lower density than water. Taking into account the absence of the dispersive solvent, it was necessary to use other methods to achieve efficient emulsification. Among the different possibilities, ultrasound-vortex-assisted was tested: 5 min of vortex and 6 min in the ultrasonic bath (100 W power) allowed us to create a stable and homogeneous emulsion. Second, 10 g L^−1^ of NaCl addition was necessary to break the emulsion.

Following this scheme, 2.5 g of honey and 2 µL of phenanthrene were brought to 10 g of aqueous solution, pH 4. Then 150 µL of toluene was added, identified as the best extraction solvent. Subsequently, the sample was subjected to 5 min stirring and ultrasounds for 6 min and NaCl 10 g L^−1^ was added. The solution was centrifuged for 30 min at 4000 rpm to break the emulsion, then 1 μL was injected into the GC–IT/MS instrument. All experimental conditions were applied to study the analytical parameters of the PAE and BP-A extraction.

### 2.3. GC–IT/MS Analysis

A TraceGC gas chromatograph (GC) coupled with an ion trap mass spectrometry (IT/MS) PolarisQ (ThermoFischer, Milan, Italy) was used for the analysis. The data acquisition and process were performed by specific software (Xcalibur, version 1.4.1, ThermoFischer). A fused-silica capillary column (SE-54, 5% phenyl–95% dimethylpolysiloxane, 30 m×0.25 mm×0.25 μm; Teknokroma, Rome, Italy) was used. Helium 5.5 was used as carrier gas at flow rate of 1.0 mL min^−1^. Inside the ion trap, helium as dumping gas was flowed at a rate of 0.3 mL min^−1^.

A programmable temperature vaporization (PTV) injector in splitless mode was used: 10 s after the injection, the PTV was heated from 100 °C to 330 °C at 800 °C min^−1^; the splitter valve was opened after 150 s. The oven temperature program was 100 °C for 1 min, increased up to 330 °C at a rate of 10 °C min^−1^ and kept at this temperature for 3 min.

The acquisition was made in full scan in a range of atomic mass units between *m/z* 75 and 400 a.m.u. Selected ion monitoring (SIM) mode was used for the analysis of the different phthalate compounds (Table 1). All the samples were determined in triplicate.

## 3. Results

Among the different variants of the DLLME technique developed recently [41,42,43,44,45], the authors optimized the ultrasound-vortex-assisted dispersion liquid–liquid microextraction (UVA-DLLME). In fact, already in other studies carried out by this research group [46,47,48,49], the authors have focused their attention on the elimination of the dispersive solvent in favor of reducing the solvents used. To create an optimal analytes extraction, the vortex was used for 5 min first and then the ultrasonic bath for 6 min. By means of the reduction of the interfacial tension, this coupling helps to determine the microdispersion of the extractant in the aqueous solution obtaining an optimal size of the microdrops with consequent high extraction efficiency. The use of vortex helps the extraction solvent in the initial dispersion within the aqueous sample. Subsequently, the ultrasounds supply adequate energy for obtaining a microdispersive phase in order to achieve quantitative extraction of the molecules. The methodological evaluation was performed on solutions containing the real matrix, adding 10 g L^−1^ of NaCl to standardize the ionic strength of all the samples and to favor a better breaking of the emulsion. The quantification of the analytes for the optimization of the method was carried out by percentage comparison with a standard solution.

First, the PAE/BP-A recoveries by each organic solvent were investigated in order to determine the solvent that had the highest percentage of recovery of the analytes present in the solution. As for the extraction solvent, a volume as low as possible to reach good preconcentration factors should have been preferred, but too low volumes generate practical problems in recovery. At the same time, too high volumes decrease the system performance due to dilution problems. Among the different solvents studied (such as *n*-heptane, *n*-hexane, *iso*-octane, ethyl acetate, toluene, and toluene:ethyl acetate 1:1), toluene has allowed to obtain the best recoveries (Table 2).

**Figure 1:**
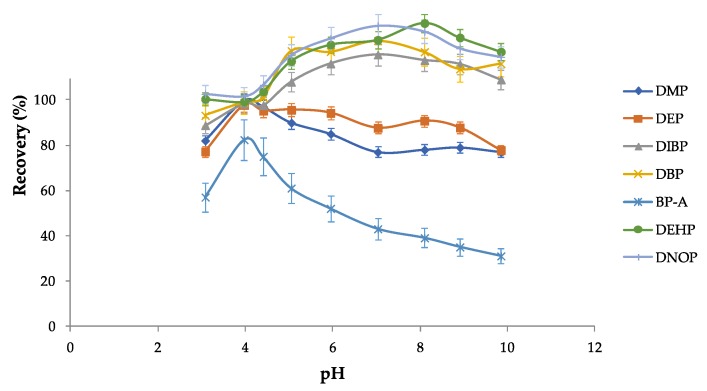
Effect of the pH on the recoveries of PAEs/BP-A investigated in this study. The conditions were as follows: each PAE/BP-A at 50 ng mL^−1^, 150 μL of toluene, 10 g L^−1^ of NaCl, and 6 min of ultrasounds.

Operative parameters for vortexing and ultrasounds were established on the basis of previous studies carried out in this laboratory [26,50]. To better optimize the PAE/BP-A recoveries, experiments were carried out with the selected solvent (toluene) by varying the pH of the aqueous solution containing the analytes. Figure 1 shows the different PAE/BP-A recoveries at different pH values; as it can be seen, the maximum extraction efficiency was at pH 4.

For determining the repeatability and the accuracy of the developed analytical method, the intraday and interday errors were studied, respectively. Table 3 shows the results obtained: intraday errors ranged between 2.2 and 11.6 whereas the interday errors ranged between 2.6 and 17.6. Further, the limit of detection (LOD) and the limit of quantification (LOQ) of each compound were investigated. In particular, LOD and LOQ are defined as the quantities of analyte that produce a signal equal to three and seven times the standard deviation of the gross blank signal, respectively [51]. Table 3 shows the limits for investigating PAEs and BP-A in honey matrix using the analytical conditions developed: LODs ranged between 2 and 6 ng·g^−1^ for the 7 PAEs and 11 ng·g^−1^ for BP-A, whereas LOQs ranged between 5–11 ng·g^−1^ for the 7 PAEs and 16 ng·g^−1^ for BP-A. It should be underlined that LODs and LOQs were directly determined on the honey matrix. These limits are sufficient for determining PAEs and BP-A in such matrices according to the SMLs reported in the regulations. Finally, the correlation coefficients were above 0.998 in the range 20–2000 ng·g^−1^. Eight calibration points were considered at 20, 50, 75, 100, 200, 500, 1000 and 2000 ng·g^−1^, directly on the honey matrix.

As some plastic can migrate from food contact materials, the EU Commission has defined the presence and the levels of small amounts of additives in food up to a food is still assumed safe for the human intake. Particularly, according to the EU Commission No. 10/2011 and 213/2018, the safety limit, defined by each SML in food, for DMP, DEP, DiBP, and DnOP is 60 mg·kg^−1^, whereas they are 0.3, 0.05, and 1.5 mg·kg^−1^ for DBP, BP-A, and DEHP, respectively. The limit of 60 mg·kg^−1^ deserves a consideration: this high value means that the additive is permitted to be used in the polymer production for food packaging and there are no restrictions provided. The LOQs are lower than the SML provided by the EU Commission: this means that the method investigated is sensitive enough to analyze the threshold limits of the different compounds in the collected honey samples.

**Figure 2:**
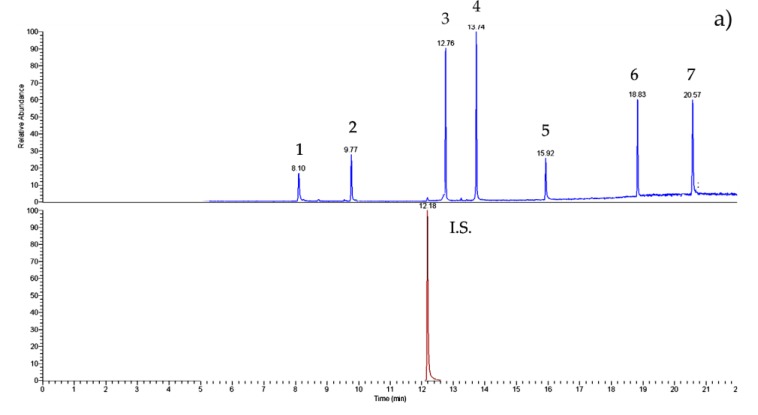

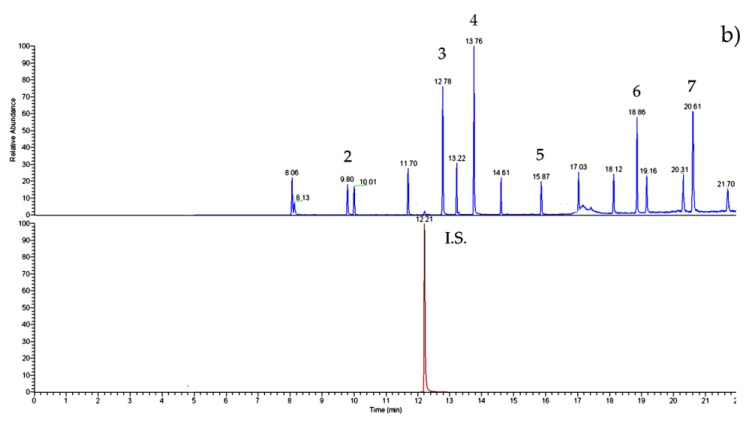
GC–IT/MS chromatograms of (**a**) PAE/BP-A (50 ng mL^−1^ of each) standard solution and (**b**) nectar honey sample (#27). For experimental conditions, see text. Peak list: 1—DMP; 2—DEP; internal standard (I.S.)—phenanthrene; 3—DiBP; 4—DBP; 5—BP-A; 6—DEHP; 7—DnOP.

Figure 2 shows the chromatograms both of the standard solution (a) and of the nectar honey sample (b). The peaks are well-solved; the PAE/BP-A determinations are precise and accurate.

It should be pointed out that no clean-up step was necessary: this occurrence was just evidenced in our previous paper on the honey matrix for determining acaricides [5] and in this case it is another confirmation.

**Figure 3:**
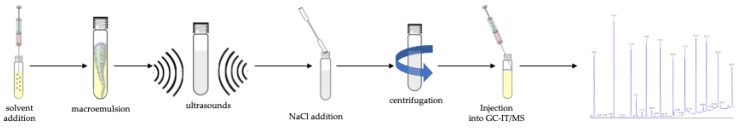
Workflow from sample preparation to data analysis of the entire analytical procedure.

Using the analytical protocol developed (briefly resuming: 10 mL of water solution at pH 4 containing 2.5 g of nectar honey sample and I.S., addition of 150 μL toluene as extraction solvent, vortex for 5 min, ultrasounds for 6 min, addition of 10 g L^−1^ of NaCl, centrifugation for 30 min at 4000 rpm, 1 μL injection into GC–IT/MS, see Figure 3; in Figure S1 the MS spectrum of each PAE and BP-A investigated in this study is reported), 47 samples of nectar honey collected in Central Italy were analyzed. The samples were collected in four different places. Table 4 reports the measurement (ng g^−1^) performed in all the samples.

As it can been seen, the DMP level was below the relative LOQ in all the samples, except in sample # 36 (12 ng g^−1^) whereas BP-A, DEHP, and DnOP showed interesting levels in some samples. Interesting levels were detected for samples #22, #33, and #35. Sample #44 showed relevant levels of BP-A and DEHP, while #45 showed relevant levels of DEHP and DnOP. Finally, the very high Pearson’s correlation coefficient, 0.880, between DEHP and DnOP, the most detected compounds, should be noted. From a toxicological point of view, these levels were always below the lethal dose (LD_50_) for each element, reported in Table 1, as well as below the relative SMLs, except for DBP and BP-A in some samples (#32 for DBP and #21, #32, #34 and #43 for BP-A). It should be underlined that the samples #32 and #34 showed the highest DBP and BP-A concentration levels of all the samples analyzed: the reason could be due to the presence of a large industrial area in the proximity of both beehives. Over the SMLs, it should be also taken into account the octanol–water partition ratio (K_ow_) of each compound: it is the most common way of expressing the lipophilicity of a compound, and it is defined as the ratio of the concentration of a solute in a water-saturated octanolic phase to its concentration in an octanol-saturated aqueous phase [52]. A high octanol–water partition ratio therefore indicates a highly lipophilic substance, whereas one with a very low coefficient (<< 1) indicates a highly hydrophilic substance. The K_ow_ values can be directly used for the evaluation of the potential bioaccumulation: according to the screening assessment for organic compounds with a K_ow_ < 4.5, it is considered that the affinity for the lipid layer of an organism is such that the substance is not considered bioaccumulative. In this case, DMP, DEP, DiBP, DBP, and BP-A showed coefficients below 4.5 whereas DEHP and DnOP coefficients were above 4.5 (7.27 and 8.10, respectively). Luckily, the LD_50_ of DEHP and the SML of DnOP are very high (10,000 mg kg^−1^ and 60 mg kg^−1^, respectively). These considerations mean that the PAE presence in the 47 samples (and the relative concentration levels) should not be of concern from a health point of view.

**Figure 4:**
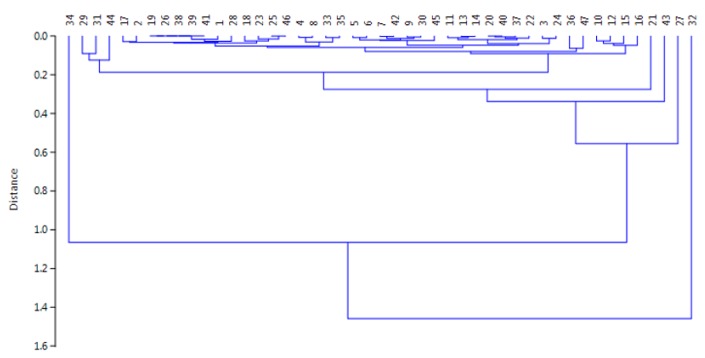
Dendrogram showing the similarities among the samples investigated in this study.

For a deeper knowledge about the correlations among the different samples, a chemometric approach was tested based on the Hierarchical Cluster Analysis (HAC) and the Principal Component Analysis (PCA) for evidencing eventual similarities. The method was applied to overall the data by the use of the Tanagra open-source software [53], by means of the centroid merge method and the Euclidean distance as a proximity measure [54,55,56]. First, the HAC evidenced the presence of four different clusters: two formed by one sample (cluster 1 by sample #32 and cluster 2 by sample #34), one by two samples (cluster 3 by samples #27 and #43), and the fourth by 43 samples (i.e., 91.5% of overall samples). Figure 4 shows the dendrogram and the relative similarities among samples.

**Figure 5:**
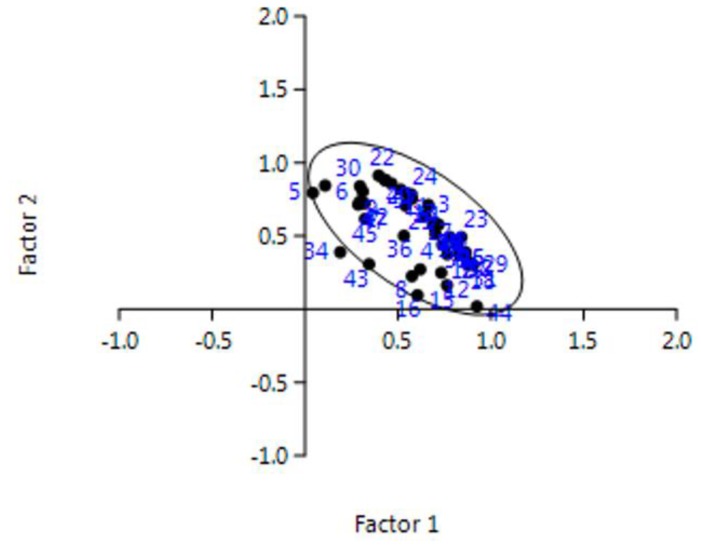
Factor analysis applied to overall data. For the F1 and F2 meaning, see the text.

Starting from this consideration, the authors applied the PCA to the data. First, three parameters (i.e., BP-A, DEHP, and DnOP) could be considered sufficient to describe 96% of the overall data. Figure 5 shows the factor analysis applied to overall the samples. For the case under investigation, the authors determined Factor 1 (F1) and Factor 2 (F2):

F1 = 0.047 DEP + 0.228 DiBP + 0.203 DBP - 0.559 BP-A + 1.735 DEHP + 1.607 DnOP

F2 = 0.812 DEP − 0.474 DiBP + 1.376 DBP + 1.118 BP-A − 0.891 DEHP + 1.086 DnOP

**Figure 6:**
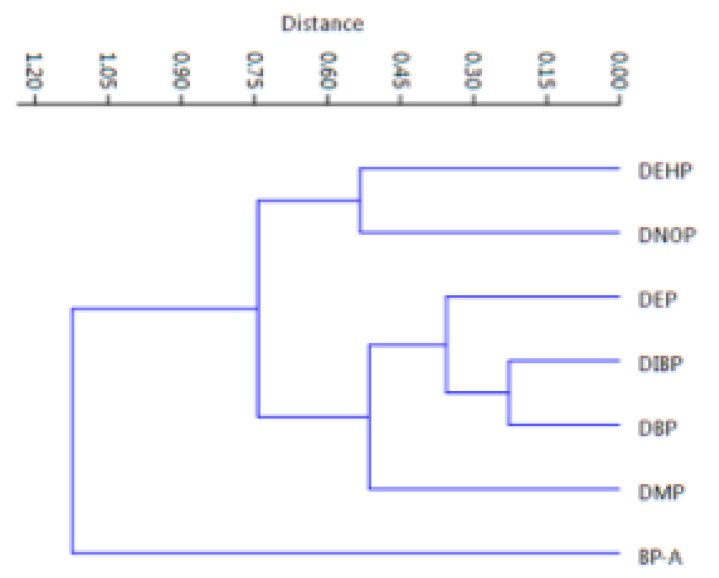
Hierarchical Cluster Analysis for evidencing the correlations among the six PAEs and the BP-A.

As it can be seen in the figure, all the samples cluster in one group, including samples #5, #16 and #43, except only the sample #34 which is out of the cluster (sample #32 is in as well as sample #27). This analysis confirms that the samples come from the same area (Central Italy), characterized by some differences which however do not affect the similarities among samples yet. Finally, Figure 6 shows how PAEs and BP-A were related between them: the six phthalates appear as a unique cluster, whereas BP-A is quite different.

This confirms the difference between these two classes of compounds both in origin and in their different widespread usage: PAEs, esters of phthalic anhydride, are mainly used as plasticizers, whereas BP-A, belonging to the group of diphenylmethane derivatives and bisphenols, is a plastic precursor, primarily polycarbonates and epoxy resins. In 2015, the global volume of BP-A consumption was estimated at 7.7 million tons, making it one of the highest volumes of chemicals produced worldwide. It is forecast to be 10.6 million tons in 2022, at an annual growth rate of almost 5% [57].

## 4. Conclusions

An increasing amount of plastic is ending up on our plates. Consumers do not notice it because they are very small particles, between 1 nm and 5 mm, called microplastics or nanoplastics. Their effects on human health are now not quantifiable. They derive from waste and, through various paths, they enter the food chain and go as far as food. Recent studies [58] have shown the wide scope of the phenomenon. It is wrong to think that fish is the only contaminated food. In 19 honey samples, taken in Germany, France, Italy, Spain, and Mexico, microplastics were found [59] and the sources are still unknown. This paper evidences plastic residues in nectar honey samples measuring different compounds characterizing such material, i.e. phthalates and bisphenol A. Although their presence and performance in plastics are well-studied, their determination is not a trivial issue: the authors developed a rapid and accurate method based on UVA-DLLME and GC–IT/MS for analyzing 7 among them (i.e., DMP, DEP, DiBP, DBP, BP-A, DEHP, DnOP). It should be underlined that the proposed method does not require any clean-up procedure as evidenced in a previous paper on the honey matrix. The entire protocol, validated for all the analytical parameters, was applied to the determination of such residues in 47 nectar honeys sampled: a chemometric approach allowed us to demonstrate both how all the samples clustered in a single group, except one sample, and how the PAEs came from the same source whereas BP-A showed a different provenance.

## Figures and Tables

**Table 1:** Chemical and legislation data of the compounds investigated in this study.

Compound	Abbreviation	CAS ^1,#^	MW ^2^	SIM ^3^	K_ow_^4^	LD_50_^5^	SML ^6^
Di-methyl phthalate	DMP	131-113	194.18	163, 194	1.60	44.7	60
Di-ethyl phthalate	DEP	84-66-2	222.24	149, 177	2.42	15.1	60
Di-isobutyl phthalate	DiBP	84-69-5	278.34	149, 223	4.11	2.04	60
Di-n-butyl phthalate	DBP	84-74-2	278.34	149, 205	4.50	1.69	0.3
Bisphenol A	BP-A	80-05-7	228.29	213, 228	3.32	35.26	0.05
Bis(2-ethylhexyl) phthalate	DEHP	118-81-7	390.56	149, 167	7.27	10000	1.5
Di-(n-octyl) phthalate	DnOP	117-84-0	390.56	149, 279	8.10	4.73	60

^1^ CAS: Chemical Abstracts Service; #: CAS number; ^2^ MW: molecular weight; ^3^ SIM: selected ion monitoring; ^4^ K_ow_: octanol–water partition coefficient; ^5^ LD_50_: lethal dose (mg kg^−1^); ^6^ SML: specific migration limit (mg kg^−1^).

**Table 2:** Recoveries (% ± standard deviation) related to the effect of different solvents on nectar honey samples spiked with a mixed PAE/BP-A standard solution. The conditions were as follows: each PAE/BP-A at 50 ng mL^−1^ and 150 μL of extraction solvent.

Solvent	DMP	DEP	DiBP	DBP	BP-A	DEHP	DnOP
*n*-hexane	15.6 ± 3.5	65.3 ± 5.2	80.9 ± 4.8	86.5 ± 5.2	40.2 ± 7.1	92.8 ± 5.2	99.4 ± 3.2
*n*-heptane	14.7 ± 4.2	63.2 ± 4.4	78.2 ± 2.9	84.1 ± 3.9	15.3 ± 6.9	91.7 ± 3.4	97.1 ± 3.4
*iso*-octane	60.3 ± 5.1	96.6 ± 3.8	110.8 ± 4.1	138.6 ± 4.1	25.0 ± 5.7	76.0 ± 4.2	76.1 ± 1.9
et. acetate ^1^	76.1 ± 3.2	76.3 ± 3.7	73.9 ± 3.6	78.5 ± 3.7	51.3 ± 6.1	67.0 ± 5.1	76.2 ± 2.9
toluene	91.2 ± 3.5	88.3 ± 4.2	89.4 ± 4.7	96.1 ± 3.9	69.3 ± 6.7	98.8 ± 3.1	97.0 ± 2.9
tol+et ^1^ 1:1	112.9 ± 4.9	87.9 ± 5.1	113.9 ± 3.9	95.6 ± 5.1	65.6 ± 7.3	64.0 ± 2.9	86.0 ± 3.2

^1^ ethyl acetate.

**Table 3:** Retention time (t_r_, expressed as min), intraday and interday precision calculated as relative standard deviation (RSD) %, limit of detection (LOD) (ng·g^−1^), limit of quantification (LOQ) (ng g^−1^), and recovery (%) in blank and honey samples of each PAE and BP-A investigated in this study.

Compound	Regr. Eq. ^1^	Intraday ^2^	Interday ^2^	LOD	LOQ	Recovery ^1^	
						Blank	Honey
DMP	y = 0.616x + 0.215	2.2	2.6	6	9	97.9 ± 1.6	91.2 ± 3.5
DEP	y = 0.875x + 0.356	2.6	4.1	5	11	99.3 ± 1.2	88.3 ± 4.2
DiBP	y = 0.598x + 0.434	2.8	3.3	2	7	97.5 ± 1.9	89.4 ± 4.7
DBP	y = 0.736x + 0.197	2.8	3.9	3	8	99.5 ± 2.1	96.1 ± 3.9
BP-A	y = 0.569x + 0.283	11.6	17.6	11	16	89.4 ± 1.7	69.3 ± 7.8
DEHP	y = 0.148x + 0.610	3.6	4.8	2	5	99.3 ± 2.1	98.8 ± 3.1
DnOP	y = 0.259x + 0.532	3.5	4.2	4	10	98.9 ± 2.5	97.0 ± 2.9

^1^ Regression equation. ^2^ Repeatability and recoveries were studied spiking the honey samples with a mixture standard solution of phthalates and BP-A at concentration of 50 ng·g^−1^ each.

**Table 4:** Levels (ng g^−1^) of DMP, DEP, DiBP, DBP, BP-A, DEHP and DnOP in the 47 nectar honey samples analyzed. For abbreviations see Table 1. For LOQs see Table 3.

# Sample	DMP	DEP	DiBP	DBP	BP-A	DEHP	DnOP
# 1	< LOQ	< LOQ	< LOQ	< LOQ	24.6	65.0	< LOQ
# 2	< LOQ	19.9	< LOQ	< LOQ	18.8	< LOQ	< LOQ
# 3	< LOQ	< LOQ	< LOQ	< LOQ	22.6	18.6	< LOQ
# 4	< LOQ	< LOQ	< LOQ	< LOQ	23.1	< LOQ	6.9
# 5	< LOQ	< LOQ	< LOQ	< LOQ	< LOQ	6.7	5.1
# 6	< LOQ	< LOQ	< LOQ	< LOQ	< LOQ	5.7	10.7
# 7	< LOQ	< LOQ	< LOQ	< LOQ	< LOQ	25.8	15.1
# 8	< LOQ	< LOQ	< LOQ	< LOQ	31.0	< LOQ	10.2
# 9	< LOQ	< LOQ	< LOQ	< LOQ	< LOQ	35.4	41.0
# 10	< LOQ	< LOQ	< LOQ	< LOQ	< LOQ	94.9	< LOQ
# 11	< LOQ	< LOQ	< LOQ	< LOQ	< LOQ	20.7	< LOQ
# 12	< LOQ	< LOQ	< LOQ	< LOQ	< LOQ	113.5	38.5
# 13	< LOQ	< LOQ	< LOQ	< LOQ	< LOQ	28.4	< LOQ
# 14	< LOQ	< LOQ	< LOQ	< LOQ	< LOQ	30.9	< LOQ
# 15	< LOQ	< LOQ	< LOQ	< LOQ	< LOQ	141.6	43.7
# 16	< LOQ	< LOQ	< LOQ	< LOQ	< LOQ	127.0	7.3
# 17	< LOQ	25.4	< LOQ	< LOQ	< LOQ	41.2	< LOQ
# 18	< LOQ	< LOQ	< LOQ	< LOQ	< LOQ	82.6	< LOQ
# 19	< LOQ	< LOQ	< LOQ	< LOQ	< LOQ	< LOQ	< LOQ
# 20	< LOQ	< LOQ	< LOQ	< LOQ	< LOQ	8.1	< LOQ
# 21	< LOQ	91.4	137.5	166.0	170.4	132.3	170.7
# 22	< LOQ	< LOQ	< LOQ	< LOQ	35.4	4.9	< LOQ
# 23	< LOQ	< LOQ	< LOQ	< LOQ	29.8	60.1	71.3
# 24	< LOQ	< LOQ	< LOQ	< LOQ	23.2	6.3	< LOQ
# 25	< LOQ	< LOQ	< LOQ	< LOQ	28.5	< LOQ	< LOQ
# 26	< LOQ	< LOQ	< LOQ	< LOQ	< LOQ	< LOQ	< LOQ
# 27	< LOQ	29.7	299.6	270.1	30.8	363.3	343.3
# 28	< LOQ	< LOQ	40.3	48.2	< LOQ	62.4	68.8
# 29	< LOQ	< LOQ	< LOQ	19.4	< LOQ	151.4	206.1
# 30	< LOQ	< LOQ	< LOQ	< LOQ	< LOQ	13.7	24.8
# 31	< LOQ	< LOQ	28.7	56.8	< LOQ	147.8	124.8
# 32	< LOQ	371.5	553.1	550.7	54.1	960.0	888.2
# 33	< LOQ	< LOQ	< LOQ	< LOQ	11.5	47.4	32.6
# 34	< LOQ	35.9	89.2	180.0	996.8	502.8	94.1
# 35	< LOQ	< LOQ	< LOQ	< LOQ	24.1	51.8	31.7
# 36	12.0	< LOQ	< LOQ	< LOQ	< LOQ	56.9	19.5
# 37	< LOQ	< LOQ	< LOQ	< LOQ	< LOQ	13.1	< LOQ
# 38	< LOQ	< LOQ	< LOQ	< LOQ	< LOQ	< LOQ	< LOQ
# 39	< LOQ	< LOQ	< LOQ	< LOQ	< LOQ	< LOQ	< LOQ
# 40	< LOQ	< LOQ	< LOQ	< LOQ	< LOQ	8.3	< LOQ
# 41	< LOQ	< LOQ	< LOQ	< LOQ	< LOQ	< LOQ	< LOQ
# 42	< LOQ	< LOQ	< LOQ	< LOQ	< LOQ	23.6	14.0
# 43	< LOQ	< LOQ	< LOQ	< LOQ	314.3	235.1	< LOQ
# 44	< LOQ	< LOQ	< LOQ	< LOQ	23.2	251.1	124.1
# 45	< LOQ	< LOQ	< LOQ	< LOQ	27.9	29.2	5.1
# 46	< LOQ	< LOQ	< LOQ	< LOQ	29.1	< LOQ	< LOQ
# 47	14.0	< LOQ	< LOQ	< LOQ	37.0	35.3	41.0

## Supplementary Materials

The following are available online at https://www.mdpi.com/1660-4601/17/5/1692/s1, Figure S1: Mass spectra of (a) DMP, (b) DEP, (c) DiBP, (d) DBP, (e) DEHP, (f) DnOP, and (g) BP-A.
