# Supplementary material for: Analytical Method Development and Chemometric Approach for Evidencing Presence of Plasticizer Residues in Nectar Honey Samples
Source: Int J Environ Res Public Health. 2020 Mar 5;17(5):1692. doi: 10.3390/ijerph17051692 (PMC7084514; doi:10.3390/ijerph17051692)
Supplement: Supplementary file 1 [file ijerph-17-01692-s001.pdf]

Analytical Method Development and Chemometric Approach for Evidencing Presence of Plasticizer Residues in Nectar Honey Samples

Ivan Notardonato [1], Sergio Passarella [1], Giuseppe Ianiri [1], Cristina Di Fiore [1], Mario Vincenzo Russo [1] and Pasquale Avino [1,*]

[1] Department of Agriculture, Environmental and Food Sciences, University of Molise, via De Sanctis, I-86100 Campobasso, Italy; ivan.notardonato@unimol.it (I.N.); sergio.passarella@studenti.unimol.it (S.P.); g.ianiri@studenti.unimol.it (G.I.) ; c.difiore@studenti.unimol.it (C.D.F.); mvrusso@unimol.it (M.V.R.)

* Correspondence: avino@unimol.it; Tel.: +39-0874-404-631

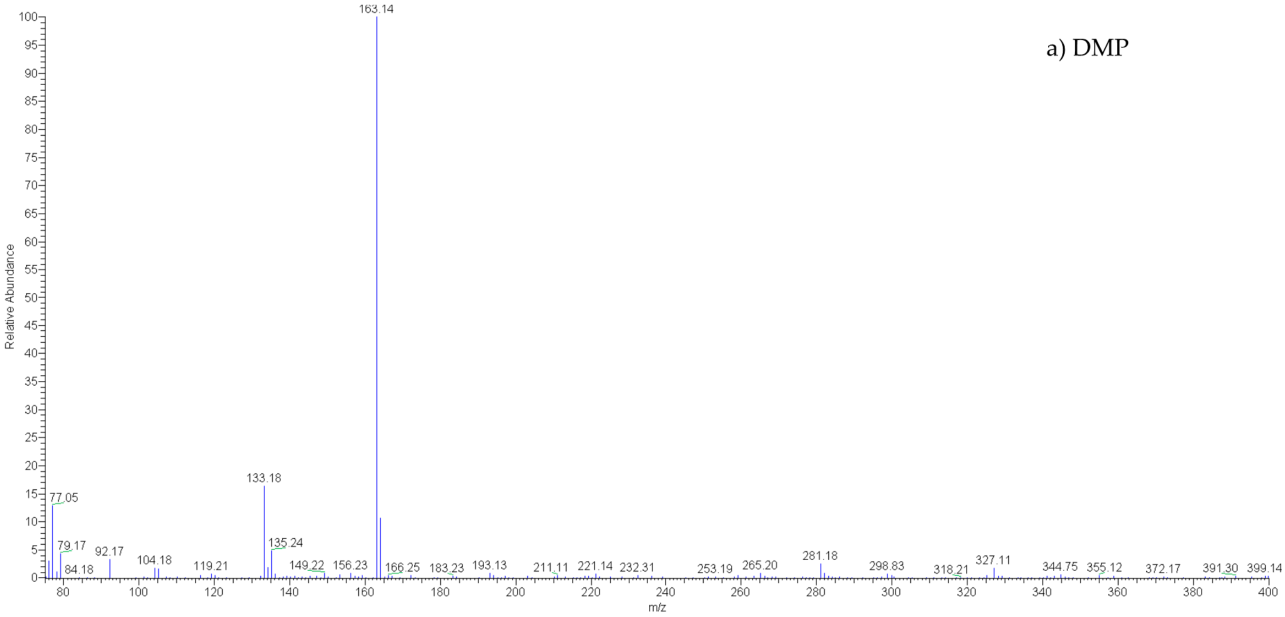
a) DMP
Relative Abundance
m/z
163.14
133.18
77.05
79.17
84.18
92.17
104.18
119.21
135.24
149.22
156.23
166.25
183.23
193.13
211.11
221.14
232.31
253.19
265.20
281.18
298.83
318.21
327.11
344.75
355.12
372.17
391.30
399.14

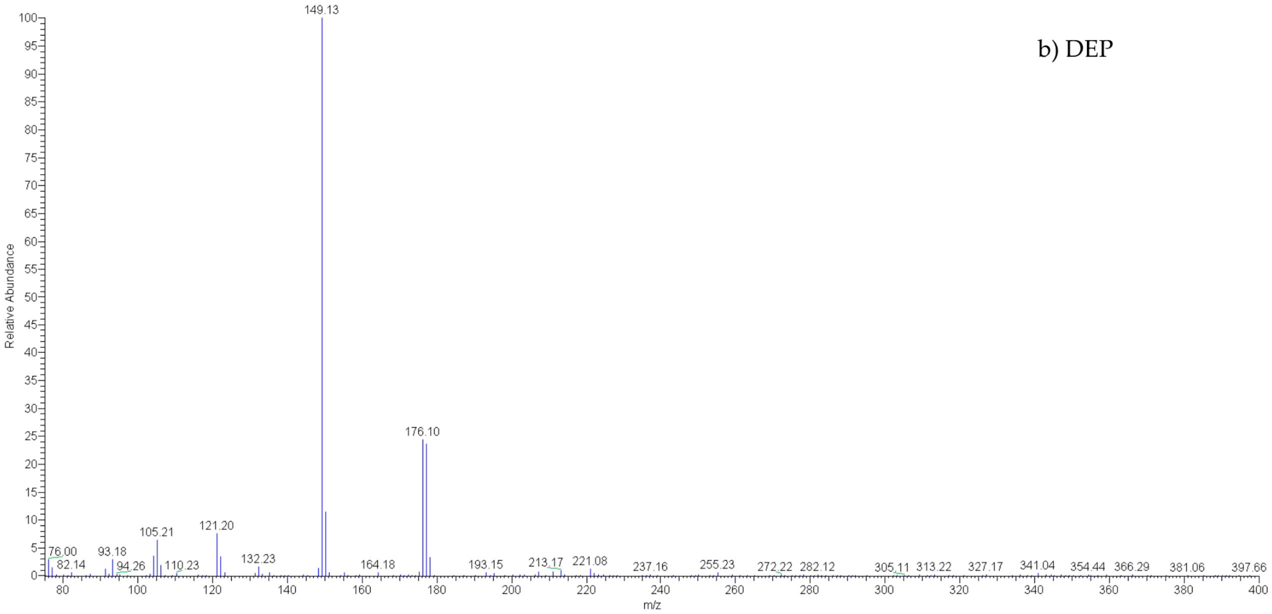
b) DEP
Relative Abundance
m/z
149.13
176.10
76.00
82.14
93.18
94.26
105.21
110.23
121.20
132.23
164.18
193.15
213.17
221.08
237.16
255.23
272.22
282.12
305.11
313.22
327.17
341.04
354.44
366.29
381.06
397.66

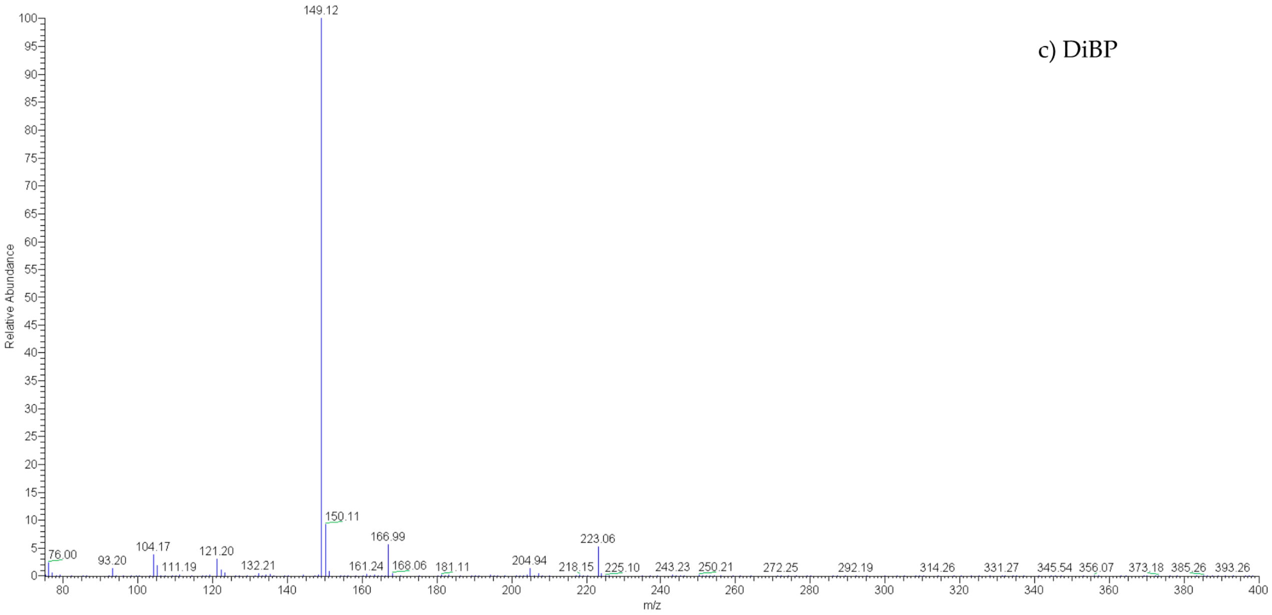
c) DiBP
Relative Abundance
m/z
149.12
150.11
166.99
223.06
76.00
93.20
104.17
111.19
121.20
132.21
161.24
168.06
181.11
204.94
218.15
225.10
243.23
250.21
272.25
292.19
314.26
331.27
345.54
356.07
373.18
385.26
393.26

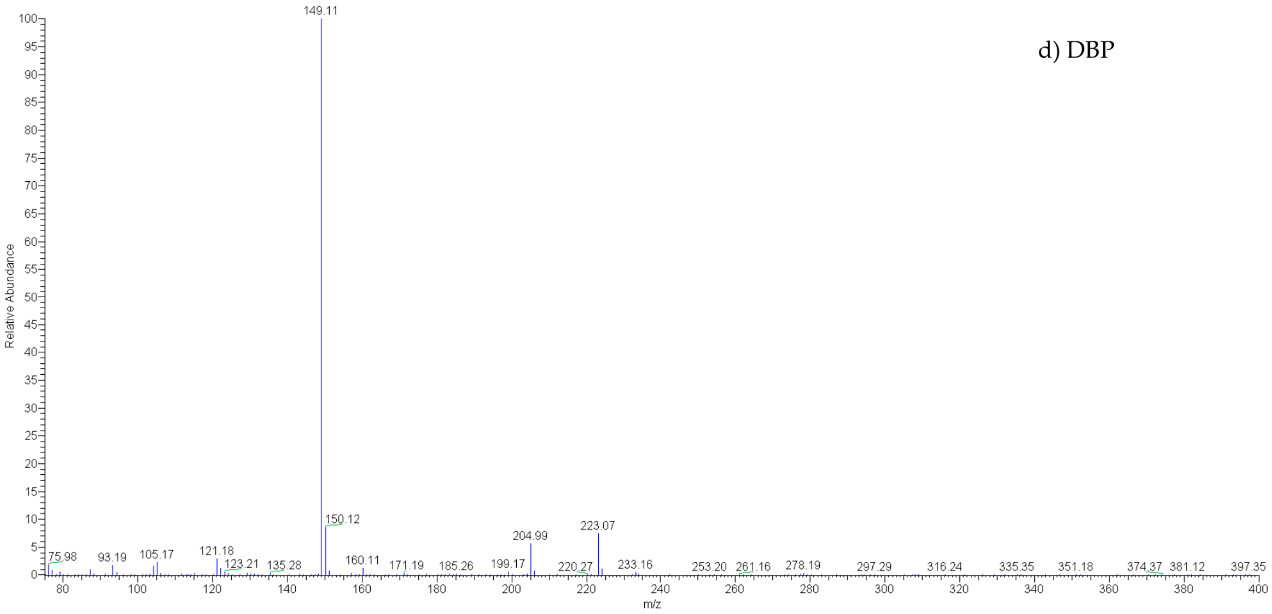
d) DBP
Relative Abundance
m/z
149.11
150.12
204.99
223.07
75.98
93.19
105.17
121.18
123.21
135.28
160.11
171.19
185.26
199.17
220.27
233.16
253.20
261.16
278.19
297.29
316.24
335.35
351.18
374.37
381.12
397.35

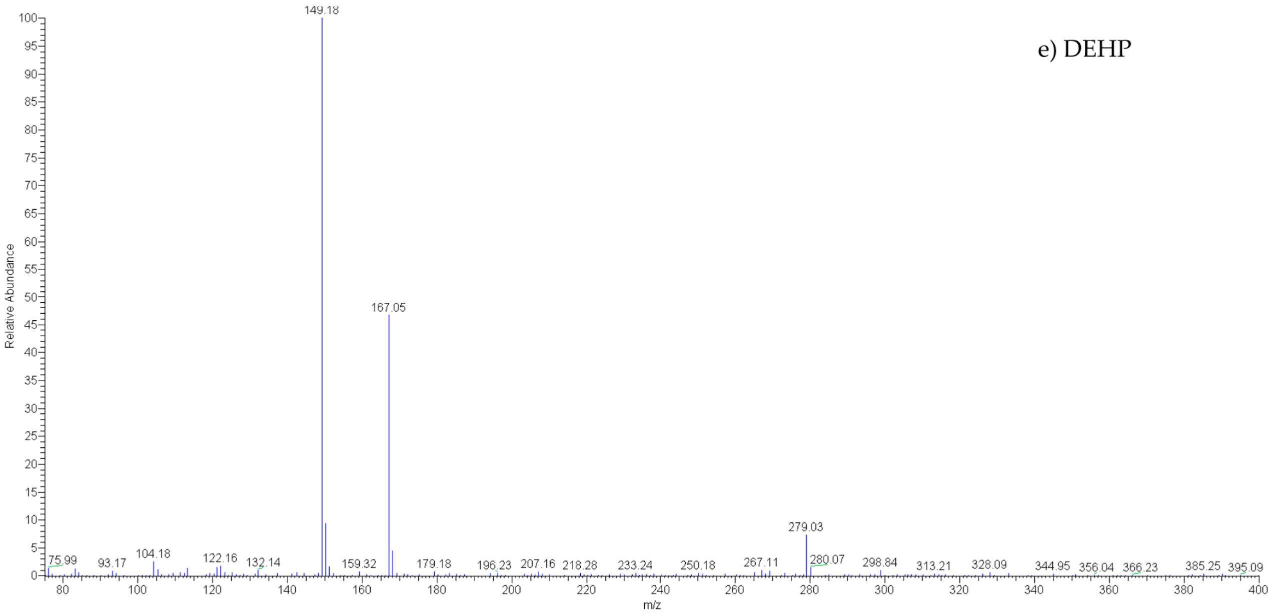
e) DEHP
Relative Abundance
m/z
149.18
167.05
279.03

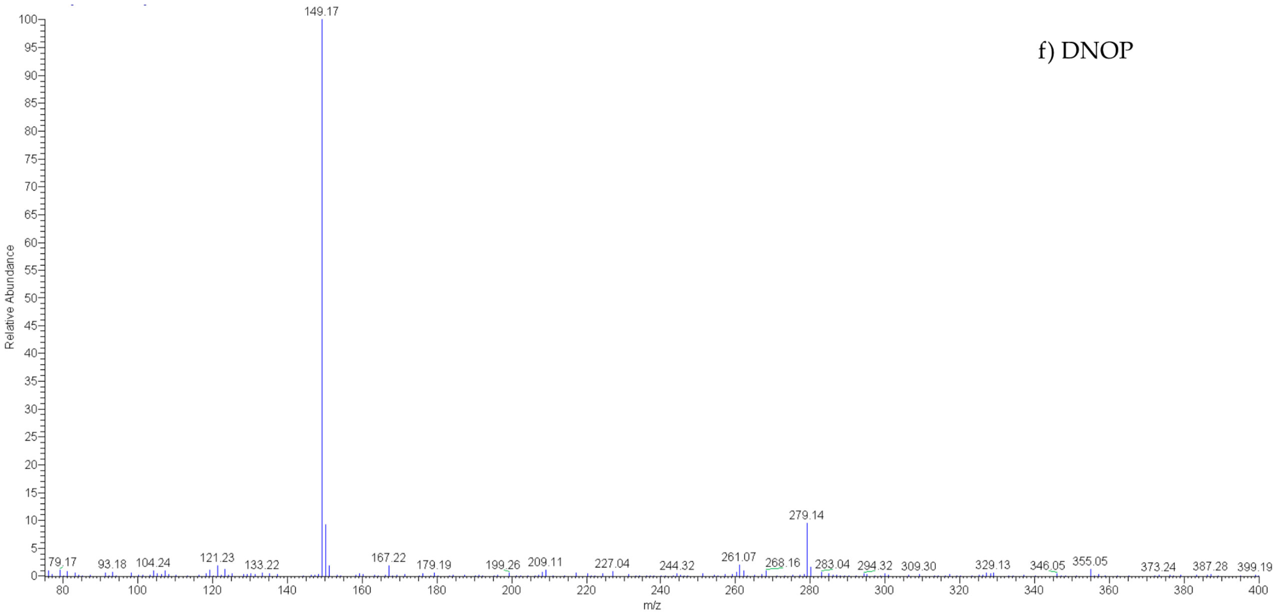
f) DNOP
Relative Abundance
m/z
149.17
279.14

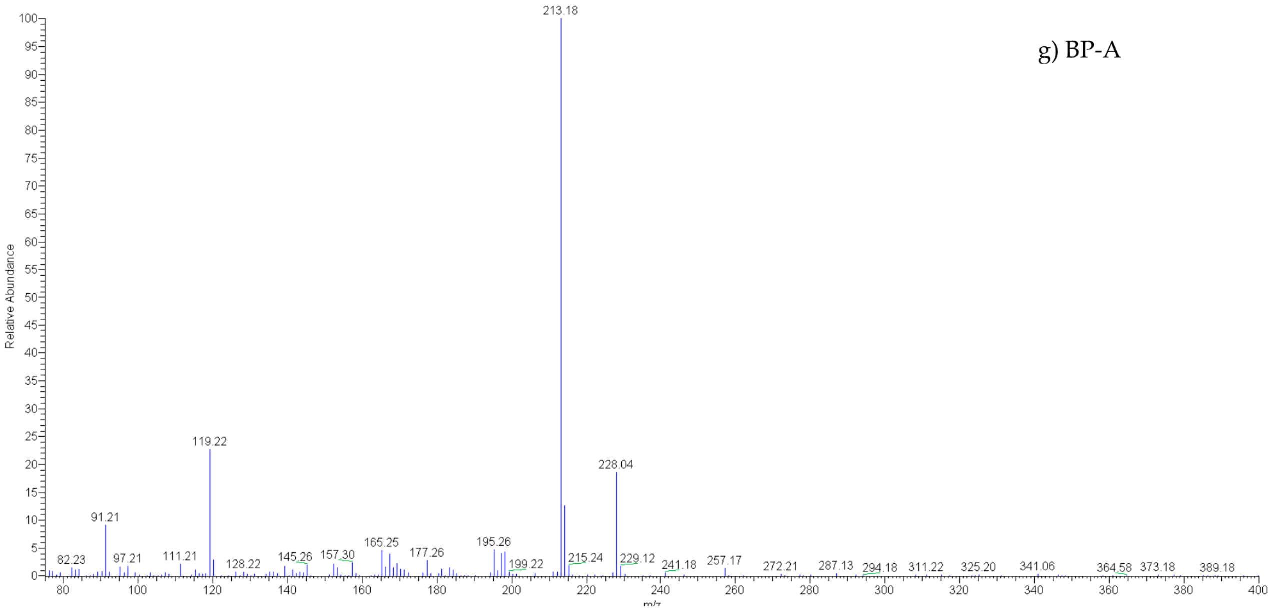

Figure S1. Mass spectra of (**a**) DMP, (**b**) DEP, (**c**) DiBP, (**d**) DBP, (**e**) DEHP, (**f**) DNOP and (**g**) BP-A.
